# Stimulating creativity in the classroom: examining the impact of sense of place on students’ creativity and the mediating effect of classmate relationships

**DOI:** 10.1186/s40359-023-01479-7

**Published:** 2023-12-07

**Authors:** Jianzhen Zhang, Yukun Yang, Jiahao Ge, Xiaoyu Liang, Zhenni An

**Affiliations:** 1 College of Geography and Environmental Science, Zhejiang Normal University, Jinhua, Zhejiang province China; 2 College of Education and Human Development, Zhejiang Normal University, Jinhua, Zhejiang province China

**Keywords:** Sense of place, Classmate relationships, Creativity, School psychology

## Abstract

**Background:**

Although previous studies have found a close relationship between sense of place and creativity, few studies have been conducted considering the micro-environment of the classroom. The mediating role of classmate relationships in the association between students’ sense of place and creativity remains unclear.

**Methods:**

This study explores classmate relationships as a mediating factor in the relationship between sense of place and creativity. Therefore, we considered a sample of 1555 Chinese high-school students and used a paper-based questionnaire survey. Data analysis was performed using SPSS 24.0, PROCESS 3.2 plugin, and AMOS.

**Results:**

Sense of place in the micro-environment of the classroom has a significant positive predictive effect on creativity. Sense of place also has a significant positive predictive effect on peer relationships. The mediation analysis reveals that peer relationships play a mediating role in the relationship between the sense of place and creativity.

**Conclusions:**

This study revealed the associations between sense of place, classmate relationships, and creativity. Creativity is better expressed in students with a strong sense of place in the classroom. Moreover, a student’s sense of place can enhance their creativity by influencing their peer relationships. These findings enrich the research in educational psychology within the classroom, providing new insights for fostering creativity.

## Introduction

Creativity promotes the development of science and technology in modern society [1], and cultivating creativity in the school context has become a core objective in many countries [2]. Creativity is typically defined as the ability to produce novel and applicable ideas or products [3]. For students, creativity is the ability to generate novel and potentially useful ideas or solutions to problems. It involves fostering the creative process and actions through experiential approaches [4], enabling students to exhibit characteristics such as novelty, flexibility, and precision in their learning [5], ultimately becoming self-directed lifelong learners [6]. Research has found that creative students are capable of solving real-world problems and achieving academic accomplishments [7, 8]. Other studies have shown that the development of students’ creativity is related to their lifelong development, including career choices, personal traits, and more [9, 10].

Given the indispensable significance of creativity in modern society [11], extensive attention has been paid to factors that influence creativity. Previous studies have highlighted that individual factors, such as gender and motivation significantly contribute to creativity [12, 13]. Additionally, external factors, such as classroom structure and atmosphere, and teachers’ attitudes toward creativity are closely related to cultivating creativity [14, 15]. The 4P model of creativity [16] and the 5 A framework of creativity [17] posit that the environment is an essential determinant of creativity. The classroom environment plays a pivotal role in supporting creativity [18] such that student creativity can be nurtured by creating a stimulating classroom environment [19]. Furthermore, sense of place, as a product of an individual’s interaction with their environment, has been demonstrated to have a direct influence on creativity [20, 21]. However, research has primarily concentrated on the relationship between individuals and macro-level environments [22–24], and less attention has been paid to the impact of sense of place in the classroom on student creativity. Therefore, it is crucial to focus on developing a stimulating classroom environment that fosters student creativity.

Classmate relationships have been identified as a crucial aspect of social cohesion in the classroom, and is defined by mutual support, care, and solidarity [25]. In the school environment, positive classmate relationships play a critical role in fostering creativity [26]. Conversely, evidence suggests that negative classmate relationships can hinder creativity by reducing the motivation to communicate [27]. In addition, Sebanc et al. [28] revealed a bi-negative relationship between negative friendships and secondary school academic achievement. Some studies have found that negative friendships can adversely affect psychological well-being [29, 30]. For example, children with negative friendships experience lower life satisfaction and higher levels of depressive symptoms [31]. Strong classmate relationships can promote collaborative knowledge sharing, enabling students to work creatively in teams [32]. Moreover, students’ sense of place in the classroom can be a driving force for emotional connection and engagement with the classroom environment, thereby facilitating classmate relationships [33]. Nevertheless, research exploring the mediating effect of classmate relationships on the relationship between sense of place and creativity is limited.

Therefore, to investigate how to stimulate student creativity in the classroom environment, this study explored the relationship between sense of place and creativity and examined the mediating effect of classmate relationships. This study not only expands our understanding of the 5 A framework of creativity but also further enriches the avenues for enhancing student creativity in the school education context.

## Theoretical basis and hypotheses

### Theoretical framework

Guilford [3] introduced the definition of creativity that included problem-solving ability, creative thought processes, innovative approaches, and the advancement of knowledge within specific academic or practical domains. Student creativity is conceptualized as the “novel and meaningful thinking that emerges during the internalization and externalization phases of learning.” [34]This study explored the development of student creativity from a theoretical framework of creativity. However, diverse theoretical frameworks for creativity have been proposed [35], shifting the focus from a singular dimension to a multidimensional perspective, and from an individual level to a sociocultural contextual level [36].

A well-accepted model of creativity introduced by Rhodes [16] is the 4P model of creativity, which comprises the dimensions of person, process, product, and place. This framework explores the personality of creative individuals, factors fostering creative environments, and the creative process. The environment can interact with external motivations to influence an individual’s creative process [37]. For instance, Yang et al. [38] found that students’ perceptions of their creative learning environment are closely linked to their scientific creativity, making the learning environment a significant predictor of divergent scientific thinking.

Recently, researchers extended the 4P model to the 5 A framework of creativity, broadening its sociocultural perspective of creativity Glaveanu [17] introduced the actor (creator), action (creative process), artifact (creative product), audience, and affordances as elements of the creative environment. The 5 A framework of creativity emphasizes specific environmental factors related to creativity. Audience refers to the social environment involved in the creative process, which includes collaborators, judges of creative products, and users. Affordances encompass the physical environment involved in the creative process, including the environment that stimulates creativity and the material conditions required for creativity. For instance, providing a harmonious learning environment in schools is one of the critical factors in fostering creativity [39]. Furthermore, a significant correlation has been found between a creative learning environment and students’ creativity through enhanced knowledge sharing, as the classroom environment provides flexible spaces and resources for students to unleash their creative potential [40].

Based on the 5 A framework of creativity, this study focused on student creativity within the classroom environment and examined the interaction between students and the physically and socially complex classroom environment to determine whether it affects the development of student creativity. The unique sociocultural perspective of the 5 A framework of creativity emphasizes attention to the individuals in the classroom, the classroom environment, and the relationship between the two. Therefore, guided by the 5 A framework of creativity, this study investigated the relationship between students’ sense of place within the classroom environment and their creativity, while also examining the mediating role of peer relationships.

### Sense of place and creativity

Sense of place, originally defined by geographer Yi-Fu Tuan [41], encompasses a universal emotional connection fulfilling people’s basic needs, whereas Relph [42] theorized it as a unique interpersonal relationship derived from an individual’s genuine emotions and authentic experiences within an environment. A sense of place broadly represents the comprehensive connection between individuals and specific locations [43], including place attachment, place identity, and place dependence [44, 45]. Place attachment signifies the emotional bond individuals have with geographic locations [46, 47]. Place dependence refers to the functional reliance on amenities and resources provided by places [48, 49]. Place identity, as defined by Proshansky [50], is a dimension of self-identity associated with an individual’s personal relationship with the physical environment. A sense of place plays a pivotal role in an individual’s overall development [51]. It is a foundation for cultivating civic character, fostering a sense of responsibility, and motivating proactive problem-solving [52]. Furthermore, there is a profound connection between sense of place and spatial cognition, in that human interactions with their surroundings and processing spatial information ultimately guide human behavior in an adaptive manner [53]. In summary, sense of place, an experiential construct generated by places and attributed by individuals, encompasses the emotional, cognitive, and attitudinal relationships between humans and places, representing the interplay of emotion, cognition, and attitude within the human–environment dynamic [54].

Furthermore, the measurement of sense of place has exhibited diverse characteristics [55]. Some researchers have developed quantitative measurement approaches for sense of place, such as the Locational Identity Scale [56] and scales assessing the seven methods of perceiving place [57], to quantify the intensity of sense of place. Qualitative research methods, including in-depth interviews and participant observation, have also been employed [58–60]. Recently, the mixed-methods approach has gained popularity [61]. This study adopted the survey items for sense of place from Jorgensen and Stedman’s [62] questionnaire, resulting in the development of a 12-item scale comprising three dimensions: Place identity, place attachment, and place dependence.

The cultivation and development of creativity are influenced by various factors, and there are noticeable individual differences in creativity [63]. For example, there is an association between gender and creativity, with women scoring higher than men on creativity assessments [64]. Additionally, living environment is related to creativity; Almeida [65] observed that living environment can provide unique stimuli for children, thereby nurturing their curiosity and creativity. The measurement of creativity is complex, and various forms and methods have been utilized, such as creative thinking tests [66] and the Ideational Behavior Scale developed by Runco et al. [67]. This study employed the Ideational Behavior Scale.

According to the 5 A framework of creativity, action, audience, and affordances are elements relevant to the development of creativity. This encourages researchers to focus on the interconnections between these elements when investigating creativity rather than isolating them. In this study, sense of place arises from the interaction between students and the classroom environment, involving the relationship between physical elements (such as decor style) and social elements (such as classroom atmosphere). These factors are closely associated with the development of student creativity [68, 69]. On the one hand, sense of place is viewed as a complex entity involving the environment and perception, with individuals with a strong sense of place being more likely to perceive the environmental characteristics of the classroom [70, 71]. For example, unique layouts and decor designs can stimulate creative thinking in students [68]. Furthermore, in terms of the classroom atmosphere, a harmonious environment provides opportunities for students to express emotions and engage in creative activities, facilitating creativity [72, 73]. In contrast, sense of place is viewed as an individual’s understanding of and emotional connection to their environment, a resource to invest in that promotes the generation and development of creativity [16]. As an emotional connection, sense of place can provide emotional support and encouragement to students [74], which also influences creativity. For instance, when students form emotional connections with the classroom environment, they receive more emotional support, which encourages their creative behaviors [75]. Therefore, based on the literature review, we proposed the following hypothesis:

#### H1

There is a positive correlation between sense of place and creativity.

### The mediating role of classmate relationships

Classmate relationships are an important form of companionship that significantly influence the development of adolescents [76, 77]. They are emotional connections that are established through specific activities among students in a learning environment [78]. The academic community generally defines the relationships between classmates as the care, support, and overall interactions experienced by students in the classroom [79]. Research has found that classmate relationships have certain effects on students’ psychosocial development, engagement in learning, and academic achievement [80, 81]. For example, the quality of classmate relationships is regarded as an important predictor of the severity of depressive symptoms in adolescents, and improving peer acceptance and reliability can enhance students’ mental well-being [82]. Good friendships can prevent conflict with peers and reduce bullying incidents, thereby promoting student engagement in learning [83].

The development of classmate relationships is influenced by various factors, such as classroom environment [84], sense of belonging [85], gender [86], and residential address [87]. The classroom environment encompasses both physical and social spaces, and constitutes a fusion of physical, social, and psychological factors [88]. Within this context, the social environment involves interactions between teachers and students as well as among students themselves [89]. A sense of place emerges from the interaction between students and environmental elements within the classroom and subsequently influences individual students and their classmate relationships [90]. Additionally, based on Maslow’s [91] hierarchy of needs, individuals have an inherent need for a sense of belonging. A sense of belonging is considered the basis for individuals to achieve self-actualization and guides them in establishing social connections [91, 92]. When students develop a sense of place in the classroom, they identify more strongly with their group, leading them to seek help from peers and cultivate positive classmate relationships [93, 94]. Gender can also influence interpersonal relationships within the classroom and is a significant factor affecting children’s friendships [95]. For example, girls are more likely than boys to exhibit prosocial behaviors, which can foster closer peer relationships [96, 97]. Finally, regarding residential location, urban students demonstrate superior interpersonal skills compared with students in rural areas [98].

The 5 A framework of creativity provides a sociocultural perspective on creativity and directs research on student creativity to focus on interpersonal relationships [99]. Enhancing the classroom environment and fostering positive student relationships can increase student creativity levels [100]. Free communication, collaboration, and positive peer relationships play an indispensable role in supporting creativity [32, 101]. Furthermore, positive relationships with classmates are crucial for developing positive learning attitudes, enhancing self-confidence, and improving judgment skills [102]. Similarly, care and support from classmates can promote student learning and subsequently stimulate their creativity [103]. The literature review revealed that friendly relationships and positive communication among peers have a significant impact on creating a healthy and harmonious environment for classroom interactions [104]. Furthermore, peer communication can foster the development of creativity [105]. Therefore, we proposed the following hypothesis:

#### H2

Classmate relationships mediate the relationship between sense of place and creativity.

**Fig. 1:**
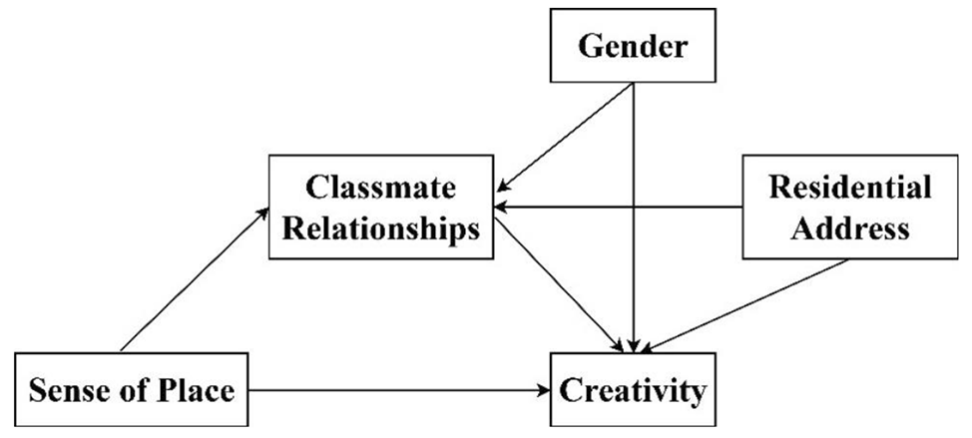
The relationships examined in the study

The hypothesized model is illustrated in Fig. 1.

## Materials and methods

### Participants and procedures

According to the principle of cluster random sampling, public high-school students from a specific region in eastern China were selected as study participants. A total of 1600 students, aged between 16 and 18, were selected. The survey was conducted using paper questionnaires, distributed from October 20 to November 20, 2022. Prior to filling out the questionnaire, the researchers explained the research content and questionnaire details to the participating students. Subsequently, with the consent of parents, homeroom teachers, and the students themselves, the researchers distributed paper questionnaires to the students and requested honest responses. Finally, the questionnaires were collected and the data were entered for analysis.

After data collection was completed, questionnaires were validated by the researchers. After excluding invalid questionnaires with missing answers, 1555 valid questionnaires were collected, resulting in a valid response rate of 97.19%. The statistical results can be found in Table 1. Among the respondents, in terms of gender, there were 746 males (47.97%) and 809 females (52.03%). In terms of place of residence, there were 899 individuals from urban areas (57.81%) and 656 individuals from rural areas (42.19%).

**Table 1:** Descriptive statistics

Variable		N	M	SD
Sense of Place		1555	57.85	12.84
	**Gender**			
	Male	746	58.28	13.28
	Female	809	57.45	12.41
	**Residential address**			
	Urban	899	58.23	12.69
	Suburban	656	57.33	13.03
Classmate relationships		1555	37.10	7.21
	**Gender**			
	Male	746	36.96	7.85
	Female	809	37.24	6.57
	**Residential Address**			
	Urban	899	37.48	6.86
	Suburban	656	36.59	7.63
Creativity		1555	41.91	10.22
	**Gender**			
	Male	746	43.51	10.88
	Female	809	40.44	9.35
	**Residential Address**			
	Urban	899	42.97	9.95
	Suburban	656	40.47	10.43

### Materials

The questionnaire consisted of two parts, comprising four sections: demographic information, sense of place questionnaire, classmate relationships questionnaire, and creativity questionnaire. In the first part, demographic information was collected, including the respondents’ gender and place of residence. The second part included the sense of place questionnaire, classmate relationships questionnaire, and creativity questionnaire. The questionnaires were all derived from the English version, and therefore, a back-translation method was employed to enhance translation quality [106].

#### Sense of place scale

The Sense of Place Scale was revised based on the Lakeshore Place Attachment survey items developed by Jorgensen and Stedman [62] in 2006. The final questionnaire comprised three dimensions and twelve items in total, namely place identity, place attachment, and place dependence. For instance, “This place defines who I am as a person” (place identity); “This place makes me happy” (place attachment); “I miss this place terribly when I am away from it” (place dependence), among others. The questionnaire uses a Likert scale of 7 points, ranging from 1 (strongly disagree) to 7 (strongly agree), with higher scores indicating higher levels of sense of place. In this study, the results of the validation factor analysis showed that the one-way model fit data were satisfactory: χ2/df = 2.51, CFI = 1.00, TLI = 0.99, RMSEA = 0.03, SRMR = 0.02. This sample exhibits good internal consistency, with a Cronbach’s alpha coefficient of 0.89.

#### Classmate relationships scale

The Classmate Relationship Scale drew inspiration from the Student Relationships Scale developed by Jiang Guangrong [107], and comprises eight question items, such as “When classmates encounter difficulties, everyone will express concern and offer help” and “Classmates support and encourage each other”. The questionnaire utilized a 7-point Likert scale, ranging from 1 (strongly disagree) to 7 (strongly agree), with higher scores indicating stronger classmate relationships. Results of the validated factor analysis showed satisfactory data for the one-way model fit: χ2/df = 2.77, CFI = 0.98, TLI = 0.98, RMSEA = 0.04, SRMR = 0.04. Cronbach’s α coefficient was calculated to be 0.76.

#### Ideational behavior scale

The Ideational Behavior Scale, developed by Runco [67], was used to measure creativity. This questionnaire comprises nine items such as, “I commonly generate novel ideas when faced with difficulties”, “I inspire classmates’ interest in innovative ideas”, “I evaluate the effectiveness of innovative ideas”, and utilizes a Likert scale of 7 points, from 1 (completely disagree) to 7 (completely agree). The higher the score, the higher the students’ creativity level. Results of the validated factor analysis showed satisfactory data for the one-way model fit: χ2/df = 2.95, CFI = 1.00, TLI = 0.99, RMSEA = 0.04, SRMR = 0.01. This sample demonstrates good internal consistency with a Cronbach’s α coefficient of 0.94.

### Data analysis

Study data analysis was conducted using SPSS 24.0 software, the PROCESS 3.2 plugin, and AMOS. Firstly, prior to data processing, Harman’s single-factor test was utilized to examine common method bias and ensure validity of the data analysis [108]. The results indicated that 28 factors had eigenvalues greater than 1, with the first factor accounting for 27.64%, which was below the critical threshold of 40%. Thus, the issue of common method bias in this study was relatively small [109]. The average values and standard deviations of the data were calculated using SPSS software, followed by the calculation of Pearson correlation coefficients to examine the relationships between sense of place, classmate relationships, and creativity. The PROCESS 3.2 plugin in SPSS was employed for mediation analysis to explore the mediating role of classmate relationships and validate the study hypotheses. In addition, CFA tests were performed using AMOS.

## Results

### Descriptive statistics and correlation analysis

A descriptive statistical analysis of sense of place, classmate relationships, and creativity is presented in Table 1. The results of the Pearson correlation analysis (refer to Table 2) revealed a significant positive correlation among the three variables.

**Table 2:** Correlation analysis

Variables	Sense of Place	Creativity	Classmate relationships
Sense of Place	1		
Creativity	0.45^**^	1	
Classmate relationships	0.44^**^	0.46^**^	1

(** = *P* < 0.01)

### Validity of measurement variables

A CFA showed that the measure model was appropriate; fit indices were acceptable: χ2/df = 2.85, CFI = 0.98, TLI = 0.98, RMSEA = 0.03, SRMR = 0.04.

### Mediation analysis

The final hypothesis of this study aims to examine the mediating role of classmate relationships. The SPSS PROCESS plugin (Version 3.2) utilizing Model 4 was employed to conduct the mediation analysis, with sense of place as the independent variable, creativity as the dependent variable, and classmate relationships as the mediating variable. Additionally, based on the literature review, gender and family residence were included as control variables. Prior to entering the mediation model, these two variables were transformed into dummy variables.

As shown in Table 3, the results indicate that sense of place has a significant positive predictive effect on creativity (β = 0.43, t = 19.40, *P* < 0.001). This prediction remains significant even after incorporating the classmate relationships variable (β = 0.29, t = 12.51, *P* < 0.001). Furthermore, sense of place has a significant positive predictive effect on classmate relationships (β = 0.44, t = 19.10, *P* < 0.001). Simultaneously, classmate relationships also have a significant positive predictive effect on creativity (β = 0.32, t = 13.65, *P* < 0.001).

**Table 3:** Mediation analysis

Regression Equation		Fitting Indices				Significance
Outcome Variables	Predictor Variables	R	R2	F(*d*ƒ)	β	T
Classmate relationships		0.45	0.20	96.39***		
	Gender				0.04	1.64
	Residential Address				-0.03	-1.22
	Sense of Place				0.44	19.10***
Creativity		0.57	0.32	145.69***		
	Gender				-0.14	-6.65***
	Residential Address				-0.04	-1.88
	Classmate relationships				0.32	13.65***
	Sense of Place				0.29	12.51***
Creativity		0.49	0.24	121.03***		
	Gender				-0.13	-5.75***
	Residential Address				-0.05	-2.18*
	Sense of Place				0.43	19.40***

* = *P* < 0.05, ** = *P* < 0.01, *** = *P* < 0.001

**Fig. 2:**
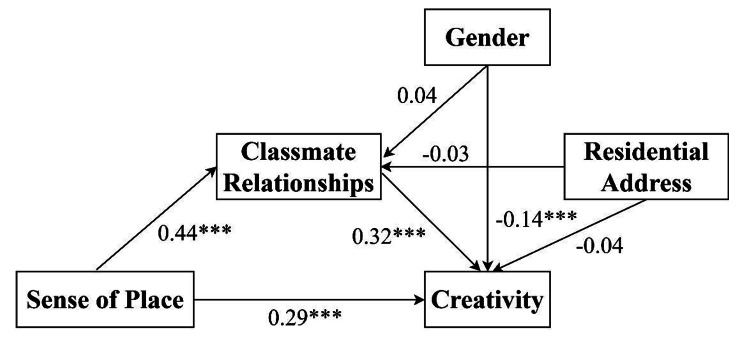
The mediation model showing relationships between sense of place and creativity, and the mediating role of classmate relationships (** = *p* < 0.01, *** = *p* < 0.001)

Based on the findings presented in Table 3, it is evident that gender influences creativity when examining the relationship between sense of place and creativity (β = -0.13, t = -5.75, *P* < 0.001). Moreover, even after incorporating classmate relationships into the model, gender continues to significantly affect creativity (β = -0.14, t = -6.65, *P* < 0.001). Figure 2 provides a graphic representation of these relationships.

In addition, the 95% confidence intervals for the direct effect of sense of place on creativity and the mediating effect of classmate relationships (see Table 4) do not include zero. This indicates that, after controlling for gender and family residence variables, sense of place can significantly predict creativity both directly and indirectly through classmate relationships. The direct effect (0.29) accounts for 67.75% of the total effect, while the indirect effect (0.14) accounts for 32.25% of the total effect.

**Table 4:** Total, direct and indirect effects among the variables

Effect	Effect Size	BootSE	BootLLCI	BootULCI	Relative Effect Size
Total effect	0.43	0.02	0.39	0.48	
Direct effect	0.29	0.02	0.25	0.34	67.75%
Indirect effect	0.14	0.02	0.11	0.17	32.25%

## Discussion

This study developed an intermediate model to explore the following hypotheses: (1) There is a positive correlation between sense of place and creativity, and (2) classmate relationships mediate the relationship between sense of place and creativity. The results support both hypotheses, as discussed below.

Our research demonstrates that sense of place is positively associated with creativity, which aligns with previous research [55, 110]. A neuroscience study found that sense of place is associated with individual behaviors, perceptions, and emotions [111], and a positive environment has been shown to enhance creativity [112]. In the context of campus settings, campus and classroom spaces bring people, ideas, and resources together [113]. Considering the campus as a living laboratory can cultivate a sense of place, enhance engagement and learning outcomes, and foster the development of research skills [114]. Furthermore, psychological evidence suggests that sensory stimuli and perceptual systems can influence creative behavior [115]. In the hippocampus, environmental changes lead to the reorganization of active neuronal ensembles or remapping of place cells [116], and brain systems and neural pathways play a crucial role in regulating creative cognition and drive [117]. The physical and sociocultural environment influences creativity [118], and the classroom space is a special environment that contains both physical and sociocultural elements. This study confirms that the sense of place that students develop within the classroom environment is conducive to creativity, further expanding the findings on creative learning environments [100, 119, 120].

### Theoretical implications

Our findings extend the 5 A framework of creativity by emphasizing the importance of focusing on environmental factors from a sociocultural perspective. Therefore, the stronger an individual’s sense of place, the stronger their attachment, reliance, and identification with the place, thereby increasing the likelihood of creative thinking and activities [121]. Autonomous motivation is a prerequisite for creativity development [122]. A close connection between classroom atmosphere and autonomous motivation, as well as a strong association between autonomous motivation and creativity, has been found [123]. Place attachment, as an emotion, is fostered when the classroom atmosphere is vibrant and students develop an attachment to the classroom, gaining a sense of place. This contributes to the student’s personal learning and engagement motivation, thereby promoting the development of creativity [124]. Thus, the study results highlight the importance of the classroom environment as a micro-level sense of place that holds significant importance for fostering creativity.

This study also found that sense of place can promote individual creativity by influencing classmate relationships; that is, classmate relationships act as a mediator. The positive association between sense of place and classmate relationships is consistent with previous research findings. A sense of place enables individuals to engage with and establish connections with the environment, thereby developing positive classmate relationships [125]. Such positive relationships require fulfilling interpersonal interactions and intimacy [126]. Having a sense of place can provide individuals with place identity, feelings of satisfaction, dependency, and a sense of belonging [127]. A sense of place can also have a positive impact on individuals’ satisfaction with their environment [128], and interactions between individuals, their environment, and others contribute to the establishment and development of trust. Mutual trust among individuals facilitates collective action, fosters a sense of collective achievement, and promotes interpersonal relationships within the collective [129]. Positive emotions experienced by students at school contribute to the formation of better interpersonal relationships [130]. The student–teacher relationship, characterized by emotional connection and respect, enhances adaptation to the classroom environment in the school setting [131, 132]. Moreover, students’ sense of place is associated with their engagement in learning. By interacting with the micro-environment of the school and classroom, students engage in teacher–student communication and interact with their peers and classmates [133]. Thus, the stronger an individual’s sense of place, the better they can integrate into the classroom environment and engage in positive interactions and communication with teachers and classmates, leading to more harmonious relationships with classmates. Therefore, it can be concluded that there is an inherent connection between sense of place and classmate relationships, which is consistent with the anticipated results of this study.

Furthermore, positive classmate relationships play a positive role in fostering creativity, which also aligns with previous research. Interpersonal relationships are a key factor influencing student creativity [134]. Several reviews and meta-analyses have concluded that positive interpersonal relationships enhance creativity and innovative behavior [135, 136]. Individuals with strong peer relationships are more likely to acquire creative thinking and behavioral patterns through peer interactions [137]. The social cognitive model [138] suggests that students’ social interactions and emotional development in the learning environment are relevant [139]. Interactions with teachers and classmates can stimulate positive learning emotions and provide opportunities for creative behavior [140]. When there are good interpersonal relationships among peers, learning emotions become more positive, and communication and interaction among classmates become more proactive, facilitating the generation and development of creative abilities. Our research findings substantiate the significance of cooperative and collaborative learning [141, 142]. High-quality classmate relationships foster teamwork and knowledge sharing, which, in turn, facilitate the development of creative teamwork among students [143].

This study demonstrated that a sense of place can influence creativity through the mediating role of classmate relationships. First, students interact with environmental elements in the classroom, facilitating the formation of place attachment, which influences their perceptions of the physical environment and interpersonal relationships within the classroom [139]. Positive emotional interactions with classmates can stimulate active thinking and foster creativity [144]. Second, neuroscientific studies have revealed that creativity is a complex psychological construct [145], and highly creative groups exhibit characteristics of collaboration and information sharing among peers [146]. School factors and classmate relationships interact to influence adolescent development [147]. In the classroom teaching environment, through the co-construction of peer engagement structures and facilitating peer discussions [148], students can effectively enhance their creative performance [149]. This study conjectures that classmate relationships, as a form of interpersonal relationships among students, can connect place attachment with creativity and serve as a mediating link between them.

### Practical implications

This study has several practical implications. First, it is crucial to recognize the impact of the physical and environmental characteristics of classrooms on students’ interpersonal relationships and intellectual development. A harmonious, positive, and nurturing classroom environment promotes students’ deep perception of the physical surroundings and emotional integration, thereby contributing to their holistic development [100]. For example, incorporating elements such as photo walls, message boards, and regular displays of students’ creative works in the classroom fosters a warm learning environment, promoting a sense of intimacy that enables students to better integrate into the class community. Student creativity is enhanced through improved interaction with the surrounding environment, peers, and teachers. Furthermore, it is necessary to emphasize the connection between social relationships, such as peer and teacher–student relationships, and students’ learning, as well as the impact of interpersonal relationships on student development. Teachers can strengthen cooperation and interaction among students by arranging seats thoughtfully and conducting group learning activities. Additionally, as a product of the interaction between individuals and their environment, a sense of place represents a unique emotional connection between students and the classroom environment. It exerts a significant influence on students’ interpersonal relationships and academic performance, highlighting the importance of emphasizing agency in students, designing student-centered activities, and paying attention to changes in students’ emotions. Moreover, the adoption of a flipped classroom approach provides students with opportunities to express their perspectives on a particular issue, enhancing student autonomy. Teachers, taking on the responsibility of guiding students through continuous brainstorming, foster increased autonomy and enthusiasm in students’ learning, thereby creating an environment conducive to the generation of more creative ideas [150].

### Limitations and future directions

Although this study has several advantages, it also has some limitations. First, this study is a cross-sectional study; thus, causal relationships cannot be determined. Second, all participants were from the eastern region of China, which limits the generalizability of the results. Third, the unequal gender ratio of the participants may also affect the generalizability of the results. In future, researchers can use a longitudinal research design to collect data over a period of time and recruit participants from different schools in different regions. Studies can focus on factors such as grade level, duration of acquaintance, and classroom atmosphere. Additionally, the researchers can examine the correlation between specific aspects of sense of place, classmate relationships, and creativity.

## Conclusions

This study focused on the relationship between sense of place and creativity in the classroom from a micro perspective, as well as the mediating role of classmate relationships. These findings have important implications for fostering student creativity in school and classroom environments as well as potential implications for their ability development and psychological well-being. This study’s innovative approach to linking sense of place and creativity in the classroom deepens our understanding of creativity and underscores the importance of classmate relationships in facilitating students’ creative potential.

## Data Availability

The datasets used and/or analysed during the current study are available from the corresponding author on reasonable request.
